# Visualization of perforin/gasdermin/complement-formed pores in real cell membranes using atomic force microscopy

**DOI:** 10.1038/s41423-018-0165-1

**Published:** 2018-10-03

**Authors:** Yuying Liu, Tianzhen Zhang, Yabo Zhou, Jiping Li, Xiaoyu Liang, Nannan Zhou, Jiadi Lv, Jing Xie, Feiran Cheng, Yiliang Fang, Yunfeng Gao, Ning Wang, Bo Huang

**Affiliations:** 1 Department of Immunology & National Key Laboratory of Medical Molecular Biology, Institute of Basic Medical Sciences, Chinese Academy of Medical Sciences, School of Basic Medicine Peking Union Medical College, Beijing, 100005 China; 2 Clinical Immunology Center, Chinese Academy of Medical Sciences, Beijing, 100005 China; 3 Beijing SOJO Electric Company Limited, Beijing, China; 4 Department of Mechanical Science and Engineering, College of Engineering, University of Illinois at Urbana-Champaign, Urbana, Illinois 61801 USA; 5 Laboratory for Cellular Biomechanics and Regenerative Medicine, College of Life Science and Technology, Huazhong University of Science and Technology, Wuhan, Hubei 430074 China; 6 Department of Biochemistry & Molecular Biology, Tongji Medical College, Huazhong University of Science & Technology, Wuhan, 430030 China

**Keywords:** membrane pore formation, AFM, perforin, GSDMD/GSDME, complement

## Abstract

Different types of pores ubiquitously form in cell membranes, leading to various types of cell death that profoundly influence the fate of inflammation and the disease status. However, these pores have never truly been visualized to date. Atomic force microscopy (AFM), which is emerging as a powerful tool to analyze the mechanical properties of biomolecules and cells, is actually an excellent imaging platform that allows biological samples to be visualized by probing surface roughness at the level of atomic resolution. Here, membrane pore structures were clearly visualized using AFM. This visualization not only describes the aperture and depth of the pore complexes but also highlights differences among the pores formed by perforin and gasdermins in tumor cell membranes and by complement in immune cell membranes. Additionally, this type of visualization also reveals the dynamic process of pore formation, fusion, and repair.

## Introduction

Membrane pore formation, an evolutionary pathway that mediates cell death, is of paramount significance in immune surveillance and pathogen clearance, as well as in the pathogenesis of inflammatory diseases^1–5^ Several executioner molecules that are capable of drilling holes in the membrane of target cells have already been identified. For instance, perforin released from T cells or NK cells forms pores in target cells at the site of the immune synapse^1,5^. Likewise, gasdermin D (GSDMD) or E (GSDME), which are activated by inflammatory caspases (caspase-1, −4, −5, and −11) or caspase-3^2,6^, form an oligomer and insert into the cellular membranes to form pores, thus mediating pyroptotic cell death. In addition, necroptotic cell death, which involves the activation of the receptor-interacting protein kinases (RIP)1/RIP3/mixed lineage kinase domain-like (MLKL) pathway, is also mediated by the formation of plasma membrane pores through MLKL^7–9^. To date, the concept of cell membrane pore formation is based on results from repeatable biological experiments and is supported by basic imaging of two-dimensional artificial liposomes using cryo-electron or other types of scanning microscopy^10–12^. However, the visualization of true pore formation has never been achieved in cellular membranes, due to the stringent requirement for high resolution (nanometer scale) and a requirement for gentle preparation methods under ambient conditions in which scanning electron microscope is usually not applicable. For example, the extremely thin sections (typically 100 nanometers) of biological specimens necessary for transmission electron microscopy are technically challenging to create. Despite the replacement of the true cell membrane, the use of liposomes for pore visualization has several limitations, including the absence of membrane proteins and the underlying cytoskeleton, leading to conceivable differences between pores in liposomes and actual cell membranes. Atomic force microscopy (AFM), a very high-resolution type of scanning probe microscopy, facilitates noninvasive imaging of cells with minimal sample preparation, where a probe is composed of a cantilever with a sharp tip mounted at its end and the probe raster scans the sample to obtain the topographic image of cells at angstrom resolution^13–15^. In this study, we report a method to directly visualize membrane pores in tumor and immune cells using AFM.

## Results

### Visualization of perforin/SLO-induced pore formation in the cell membrane

**Fig. 1:**
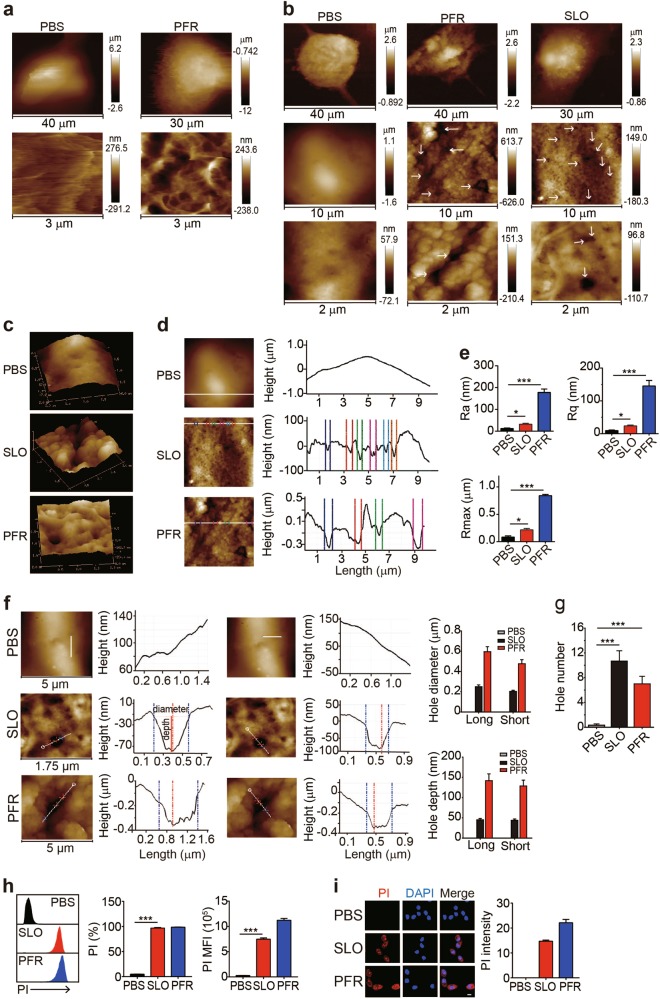
Visualization of SLO/perforin-induced pore formation using AFM. **a** OVA-B16 cells were cultured in 35 mm glass-bottom dishes and treated with PBS or perforin (PFR) (50 U) for 15 min. Cells were then washed with PBS twice and imaged under an atomic force microscope. **b–g** OVA-B16 cells (1 × 10^5^) were treated with 50 U of SLO or 50 U of PFR isolated from activated human CD8^+^ T cells for 5 min, fixed with 4% paraformaldehyde and imaged using AFM (**b**). The 3D topographies of cellular membranes are shown (**c**). Based on the high-resolution AFM topography data, a regional analysis was performed and surface roughness was analyzed (**d**). The values of Ra, Rq, and Rmax were calculated (**e**). The pore diameters along the long and short axes and the pore depth in the membranes of six OVA-B16 cells were calculated within a cellular area of 5 × 5 μm^2^ (**f**). For the diameter measurements, the white lines across the pore acted as indicator of the topographies, and the values were calculated between the two dotted blue vertical lines on the curve diagram. The pore depth was defined as the vertical distance between the two red horizontal lines on the curve diagram. Bar, 1 μm. The number of pores was quantified from three 5 × 5 μm^2^ areas from one cell (*n* = 6) (**g**). A pore was defined as a cavity in the plasma membrane with a depth greater than 10 nm. **h, i** OVA-B16 cells were treated with 50 U of SLO or PFR for 5 min. During the last 30 s of the SLO or PFR treatment, PI (100 μM) was added to the culture medium and PI^+^ cells were immediately analyzed by flow cytometry (**h**). Some cells were fixed and observed under a confocal microscope (**i**). Bar, 20 μm. The experiment was repeated three times. White arrows indicate pores. * *p* < 0.05 and *** *p* < 0.001 by one-way ANOVA **(e, g, h)**. The data are presented as the means ± SEM of three independent experiments

Upon recognizing antigens, effector CD8^+^ cytotoxic T cells (CTLs) interact with target cells such as tumor cells or virus-infected cells and release perforin and granzymes into the intercellular immune synapse, where perforin acts as an executioner to drill a hole in the membrane of target cells^16–18^. We extracted the pore-forming cytolytic protein perforin from activated CD8^+^ T cells and applied it to OVA-B16 melanoma tumor cells for 15 min to recapitulate this process. These living cells were imaged using AFM scanning. The perforin treatment induced morphological changes in living OVA-B16 cells, as evidenced by the formation of pore-like structures on the surface of the cell membrane (Fig. 1a). Living cells are very fluid and their membranes are decorated with numerous proteins, making the successful contact of the AFM probe difficult. We fixed the cells with 4% paraformaldehyde, washed the cells with PBS and scanned samples with AFM to prevent the mobility of living cells. Consistently, fixed OVA-B16 cells treated with perforin also showed the formation of pore-like structures of various sizes in the cell membrane (Fig. 1b), allowing us to use fixed cells for the experiments in this study. Additionally, we applied the bacterium-derived pore-forming toxin streptolysin O (SLO) to OVA-B16 cells for 5 min. As a result, pore-like structures were visualized in the membrane of SLO-treated cells (Fig. 1b). The three-dimensional topographies of cell membranes were obtained using AFM, further showing well-defined pore-like structures in cell membranes, as well as much rougher and more uneven cell surfaces in perforin-treated or SLO-treated OVA-B16 cells compared to the control cells (Fig. 1c, d). Consistent with these results, the values for Ra (average roughness), Rq (root mean square), and Rmax (the maximum vertical distance between the highest and lowest data points) were also markedly increased after the SLO or PFR treatment (Fig. 1e). Furthermore, we performed a metrological analysis of the diameter (long and short lengths from the horizontal surface) and depth of pore-like structures in samples using AFM NanoScope Analysis 1.8 software, which confirmed the presence of pores. In SLO-treated OVA-B16 cells, the pore diameter was 248 ± 19 nm (long length) and 202 ± 11 nm (short length), respectively, while in perforin-treated OVA-B16 cells, the pore diameter was much larger, with 594 ± 50 nm for the long length and 476 ± 40 nm for the short length (Fig. 1f). In addition, the depths of perforin-formed pores were 141 ± 16 nm from the long length and 128 ± 14 nm from the short length, while the depths of SLO-formed pores were 44 ± 3 nm or 43 ± 3 nm, respectively (Fig. 1f). These depth data excluded the possibility that the visualized pores were derived from a membrane depression or fold, because the thickness of plasma membrane of a cell is less than 10 nm. The reason why perforin formed larger holes than SLO might be attributed to the intrinsic properties and specificity of perforin for tumor cells. Furthermore, SLO and perforin formed an average of ten and seven pores, respectively, on a 5 × 5 μm^2^ membrane area (Fig. 1g). In addition to OVA-B16 cells, SLO-induced pore formation was also observed in human melanoma A375 and human liver cancer HepG2 cell lines (Supplementary Figure 1a-c). A critical trait of membrane pores is permeability. Indeed, the treated cells were effectively stained with propidium iodide (PI) (Fig. 1h, i and Supplementary Figure 1d and e). Although PI is commonly used to stain the nucleus, it is also detected in the cytosol upon binding to cytosolic RNAs^19,20^. Consistent with this finding, under our conditions, PI was actually located in both the cytosol and the nucleus. Using ultrahigh-resolution scanning electron microscopy (HITACHI SU8010), we also visualized these perforin-formed pores; however, the images are presented in a 2D format (Supplementary Figure 1f). Based on these data, SLO- and perforin-formed pores in true cellular membranes were successfully visualized using an atomic force microscope.

### Pore formation occurs with a repair process in a dose- and time-dependent manner

We used different concentrations of SLO (50, 100, 200, or 500 U) and treated OVA-B16 cells for 4 min to further analyze the effect of SLO on the pore formation process. Cells treated with 50 U of SLO rarely formed membrane pores, whereas other doses induced pore formation (Supplementary Figure 2a). Meanwhile, a dose-dependent increase in the surface roughness and increases in the Ra, Rq, and Rmax values were observed (Supplementary Figure 2b). Consistently, the size and number of pores increased as the SLO concentration increased (Supplementary Figure 2c and d). In addition, the intensity of PI staining increased as the SLO dose increased (Supplementary Figure 2e).

**Fig. 2:**
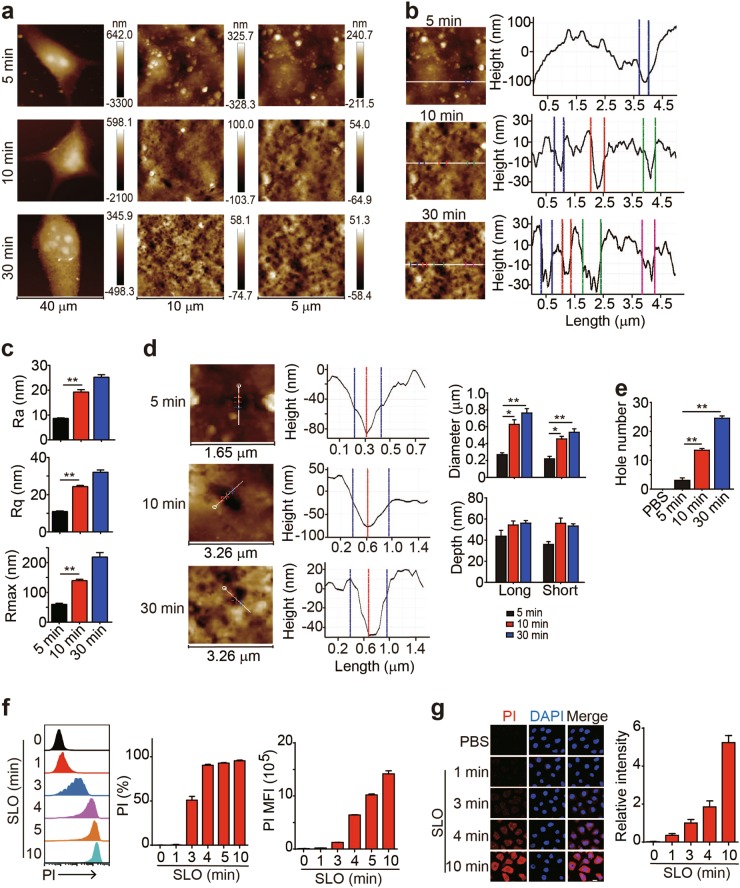
SLO-mediated pore formation occurs in a time-dependent manner. **a–e** OVA-B16 cells (1 × 10^5^) were treated with 50 U of SLO for different time intervals, as indicated. Cells were fixed and imaged using AFM. Representative images of OVA-B16 cell topographies at different magnifications are shown (**a**). Surface roughness was analyzed (**b**), and the values of Ra, Rq, and Rmax were calculated (**c**). The pore diameter and depth were measured by a section analysis of high-resolution AFM topographies (**d**). The number of pores was counted within three 10 × 10 μm^2^ areas from one cell (*n* = 6) (**e**). **f, g** OVA-B16 cells were treated with 50 U of SLO for different time points, as indicated. During the last 30 s of the SLO treatment, PI (100 μM) was added to the culture medium and PI^+^ cells were immediately analyzed by flow cytometry (**f**). Some cells were fixed and observed under a confocal microscope (**g**). Bar, 20 μm. *p < 0.05 and **p < 0.01 by one-way ANOVA **(c–e)**. The data are presented as the means ± SEM of three independent experiments

Next, we investigated the dynamic process of perforin/SLO-induced pore formation. A 4-min treatment with 50 U of SLO rarely induced pore formation in B16 cells (Supplementary Figure 2). However, when B16 cells were treated with this concentration of SLO for either 10 min or 30 min, pore formation was induced concomitant with an increase in the roughness of the cell membrane (Fig. 2a), indicating a time-dependent pore formation process. Further analyses of the cell surface showed that the cell surface became rougher with prolonged treatments (Fig. 2b), and the values of Ra, Rq, and Rmax were correspondingly increased (Fig. 2c). Moreover, a prolonged treatment time produced a greater number of pores with larger diameters (Fig. 2d, e). The formation of larger pores might be ascribed to the fusion of small pores, and SLO and perforin might continuously form pores in cellular membranes using a certain constant mode. We treated B16 cells with 50 U of SLO for the indicated time periods and added PI to the supernatant to further confirm that pore formation was a time-dependent process. PI consistently entered OVA-B16 cells in a time-dependent manner, because a 4-min treatment resulted in PI staining in cells, while a 10-min treatment markedly increased the intensity of PI staining in the cells, as evidenced by both flow cytometry and fluorescence microscopy (Fig. 2f, g). Based on these data, pore formation was a highly dynamic process, with increases in the pore size and number observed as with a prolonged treatment time.

**Fig. 3:**
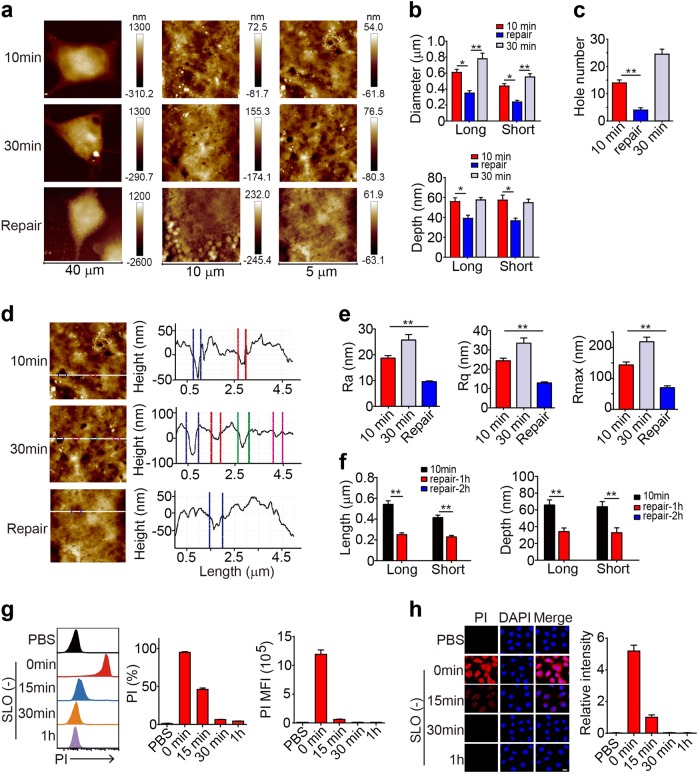
SLO-induced pore formation is repairable. **a–e** OVA-B16 cells were treated with 50 U of SLO for 10 min or 30 min. Some cells were treated with SLO for 10 min and then cultured in the absence of SLO for another 20 min. Cells were fixed and imaged using AFM. Representative images of OVA-B16 cell topographies at different magnifications are shown (**a**). The pore diameter and depth were measured (**b**). The number of pores was counted within three 10 × 10 μm^2^ areas from one cell (*n* = 6) (**c**). Surface roughness was analyzed (**d**), and the values of Ra, Rq, and Rmax were calculated (**e**). **f** OVA-B16 cells were treated with SLO (50 U) for 10 min, and then cultured in fresh medium without SLO for an additional 1 h or 2 h. Cells were imaged using AFM to determine the pore diameter and depth. (**g and h**) OVA-B16 cells were treated with SLO (50 U) for 10 min, and then cultured in SLO-free medium for another 15 min, 30 min, or 1 h, as indicated. During the last 30 s of repair, PI was added to the culture medium. PI^+^ cells were measured by flow cytometry (**g**) and confocal microscopy (**h**). Bar, 20 μm. * *p* < 0.05 and ** *p* < 0.01 by one-way ANOVA **(b, c, e and f).** The data are presented as the means ± SEM of three independent experiments

Cells might have evolved membrane repair mechanism(s) to prevent pore formation-mediated damage. Indeed, the pores induced by SLO were repaired, because the pore size, depth, and number remarkably decreased once the SLO was removed from the culture medium (Fig. 3a–c); concomitant decreases in the surface roughness and the values of Ra, Rq, and Rmax were also observed (Fig. 3d, e). Moreover, a 2-h repair caused the pores to disappear (Fig. 3f), suggesting that a dynamic process exists between membrane pore formation and repair to allow cells to repair damaged membranes. However, if the pore size exceeded 900 nm (Supplementary Figure 3a), cells were not able to easily repair the pores. OVA-B16 cells in the 200 U SLO-treated group with a 942 ± 47 nm pore size (long length) underwent death, while cells in the 50 U-treated group with a 531 ± 30 nm pore size survived well (Supplementary Figure 3b). We then further verified the pore repair process using PI staining. The SLO treatment markedly increased the intensity of PI staining in the cells, while a gradual decrease in PI staining was observed in cells after SLO removal and recuperation in new culture medium over time, and 30 min of recuperation resulted in undetectable levels of intracellular PI (Fig. 3g, h) and viable cells (Supplementary Figure 3c).

### Visualization of pore formation induced by tumor-specific CTLs

**Fig. 4:**
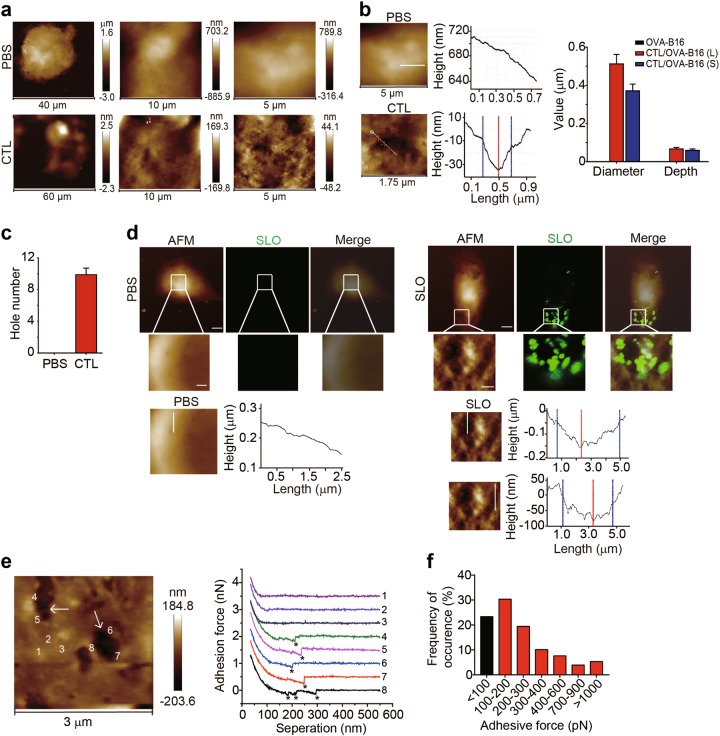
Pore formation in the membrane of OVA-B16 cells induced by tumor-specific CTLs. **a–c** Splenic CD8^+^ T cells from OT-I mice were sorted by FACS and activated by anti-CD3/CD8 beads for 48 h. Then, the T cells were incubated with OVA-B16 cells at a 20 : 1 ratio for 4 h. The suspended CTLs were removed and the remaining CTLs and OVA-B16 cells were fixed for AFM imaging. AFM topographies are shown (**a**). The diameters of the long axis or short axis and the depths of the pores were calculated by performing a section analysis of the high-resolution AFM topographies (**b**). Additionally, the number of pores that formed was counted within three 5 × 5 μm^2^ areas from one cell (*n* = 6) (**c**). **d** OVA-B16 cells were treated with PBS or biotin-labeled SLO (1000 U) and stained with Alexa Fluor 647-conjugated streptavidin. The same cell was imaged using AFM and confocal fluorescence microscopy. Using the topography from AFM and bright field images from confocal microscopy, a merged image of the cell depicting the high-resolution AFM topography and fluorescence is shown. Bars (*upper panel*), 5 μm; bar (*lower panel*), 1 μm. **e, f** OVA-B16 cells were treated with SLO (100 U) for 10 min and imaged using AFM with SLO antibody-conjugated AFM tips. Representative images of OVA-B16 cell topographies and force curves generated from the areas lacking pores and from the border of pores are presented (**e**). The distribution of the frequency of adhesion force obtained from the data points (*n* = 712) is shown (**b**). Asterisks indicate ruptures. White arrows indicate pores

Despite the aforementioned efforts to visualize the pore formation through the artificial addition of SLO/perforin, the capability of actual CTL-released perforin to drill holes remained unclear. By adapting the classical cytotoxicity assay, we cocultured activated OVA-specific CTLs and OVA-B16 cells at a 20 : 1 ratio for 4 h. Under the atomic force microscope, pore formation was clearly observed with a length of 512 ± 49 nm, a width of 370 ± 35 nm and a depth of 66 ± 8 nm (Fig. 4a, b). Ten pores formed in a cell membrane area of 5 × 5 μm^2^ (Fig. 4c). We used fluorescently labeled SLO to treated OVA-B16 cells with fluorescently labeled SLO to clarify that SLO/perforin directly induced pore formation. Cells were observed by fluorescence microscopy and AFM, respectively. Upon merging the images, pores were indeed surrounded with by SLO (Fig. 4d). We then used a comparable approach to further confirm that the pores were surrounded by SLO. Using SLO antibody-conjugated AFM tips as a probe to image SLO-treated cells, the force curves generated from the areas lacking pores did not rupture, but the curves from the border of pores had clearly ruptured (Fig. 4e), indicating that single or multiple SLO antigen-antibody bonds were formed. Moreover, we also calculated the magnitude of the adhesion force (Fig. 4f). Therefore, SLO was indeed localized around the pores. Based on these results, CTLs indeed use perforin to drill holes in the membranes of target cells.

### Visualization of GSDME/D-induced pore formation in the cell membrane

**Fig. 5:**
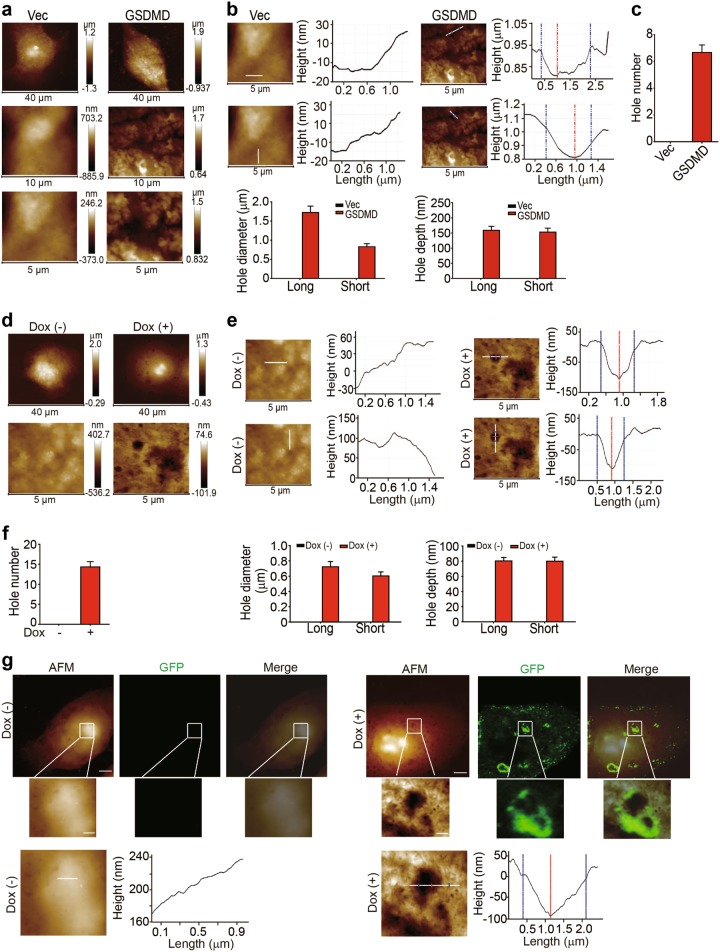
GSDMD induces pore formation in the cell membrane. **a–c** 293T cells were transiently transfected with Vec or the GSDMD^Nterm^ plasmid for 18 h. Cells were then fixed and scanned using AFM. Representative images of the topographies of the outer plasma membrane in Vec- or GSDME-transfected cells visualized by AFM (**a**). The pore diameter and depth were measured by a section analysis of high-resolution AFM topographies (**b**). The number of pores was enumerated from three 5 × 5 μm^2^ areas from one cell (*n* = 6) (**c**). **d–f** MCF-7 cells were stably transfected with the plasmid expressing GSDMD-N (L192D mutation)-eGFP under the control of a tetracycline-inducible promoter. After an 18 h treatment with or without doxycycline, cells were fixed and imaged using AFM. Representative images of the topographies of the outer plasma membrane visualized using AFM (**d**). The pore diameter and depth were measured by a section analysis of high-resolution AFM topographies (**e**). The number of pores was enumerated in three 5 × 5 μm^2^ areas from one cell (*n* = 6) (**f**). **g** The same data as shown in (**d**), except that the same MCF-7 cell was imaged using both AFM and confocal microscopy. The section analysis was performed on the high-resolution AFM topographies. Bar (*upper panel*), 5 μm; bar (*lower panel*), 1 μm

Next, we visualized gasdermin-mediated pore formation in 293T cells. GSDMD has been regarded as an executioner that mediates monocyte/macrophage pyroptosis in response to certain bacterial insults^21–23^. Both inflammasome-activated caspase-1 and intracellular lipopolysaccharide-activated caspase-11/4/5 are capable of cleaving GSDMD to generate its pore-forming active structure^21,24^. Moreover, a GSDME was recently shown to be specifically cleaved by caspase-3 and induce pyroptosis in certain GSDME-expressing cancer cells^6^. When we transduced 293T cells with the active form of GSDMD, giant pores (greater than 1 µm in diameter) were formed by GSDME (Fig. 5a–c). We transfected MCF-7 cells with a recombinant GSDMD-GFP vector containing a single nucleotide mutation in the GSDMD cDNA sequence to induce an attenuated expression pattern and further confirm our results. Approximately 14 pores formed on a 5 × 5 µm^2^ area of the cell membrane, with a diameter of approximately 0.7 µm and a depth of 80 nm (Fig. 5d–f). In addition, similar to SLO/perforin, GSDMD also encircled the pore (Fig. 5g), suggesting that GSDMD directly formed a pore in the cell membrane. Consistent results were also observed in 293T cells transduced with a plasmid containing the active form of GSDME (Supplementary Figure 4a–c). When we transduced 293T cells with the active form of GSDME, the cells exhibited a very swollen morphology (greater than 80 μm in size) and burst (pyroptosis) under the AFM (Supplementary Figure 4a). These pores were as large as 10 μm in diameter and 1.2 μm deep, reflecting cellular swelling (Supplementary Figure 4b).

### Visualization of complement-mediated pore formation in the cellular membrane

**Fig. 6:**
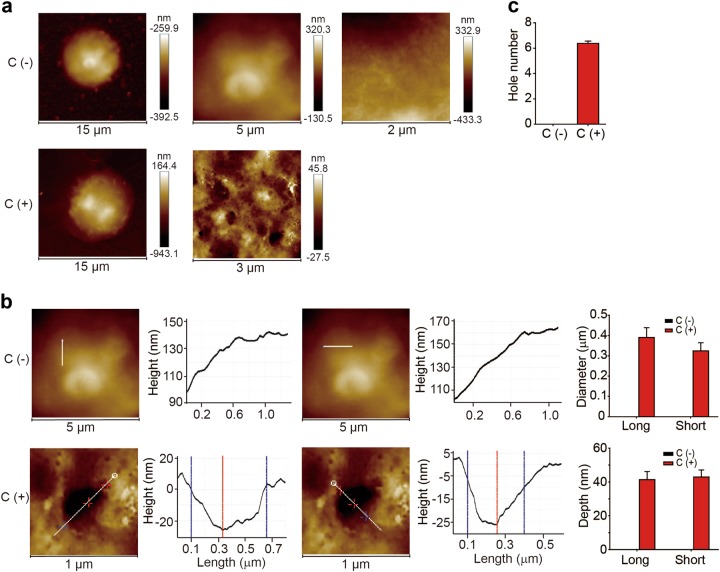
Characterization of complement-induced pore formation. After culture with IL-2 (100 U/ml) for 4 h, splenic CD4^+^CD25^+^ Treg cells were treated with an anti-CD25-depleting antibody (PC-61) or the isotype control in the presence of C57BL/6 mouse serum for 30 min. **a** Representative images of the AFM topographies of the outer plasma membrane of Treg cells. **b, c** The pore diameter and depth were measured (**b**). The pore number was calculated by analyzing three 5 × 5 μm^2^ areas from one cell (*n* = 6) (**c**)

Perforin- and gasdermin-mediated pore formation constitute an effective arm of host immune defense against transformed cells and infections. In addition to these two mechanisms, the host is also capable of mobilizing a series of serum proteins, which together comprise the complement system that mediates humoral innate immunity, leading to the formation of the membrane attack complex (MAC) by complement components C5b, C6, C7, C8, and C9^25^. In clinical practice and animal studies, complement is commonly utilized to deplete antigen-expressing cells, including immune cells, via an IgG/IgM-activated complement cascade reaction both in vitro and in vivo^26^. However, to date, complement-mediated pore formation in real cells has not been visualized. We treated regulatory T cells with an anti-CD25 depleting antibody (PC-61) in vitro in the presence of murine serum. After a 30 min treatment, pores with a diameter of 400 nm were observed in the membrane of Treg cells (Fig. 6a, b), and the pore density was estimated to be seven pores per 5 × 5 μm^2^ area (Fig. 6c).

## Discussion

AFM is an invaluable tool for obtaining high-resolution topographical images and has been used in a wide range of applications, from materials science to biology^27,28^. Unlike optical or electron microscopy, which use photons or electrons directed onto or emitted from the specimen as probes to yield information about the sample, AFM uses a sharp tip as a probe. This probe is approximately one-tenth of a nanometer in diameter and is attached to the end of a cantilever to scan the surface topography of samples. Thus, AFM exceeds the resolution of an electron microscope, reaching < 1 nm, conferring AFM with the ability to image samples ranging from atoms, molecules, and molecular aggregates to cells^29^. Cellular membranes may contain various pits. The main difference between pores and pits is that the cell membrane integrity is lost when pores form, while the cell membrane maintains its integrity when pits form. If the cell membrane only contains pits, then the membrane-impermeable dyes such as PI do not enter the cells. In the present study, we excluded the possibility that pits formed by performing PI staining.

AFM has been applied to image proteins, DNA, viruses and other biological samples^28,30,31^; however, AFM has not been used to image pores in true cell membranes in previous studies. In the present study, we used the PeakForce Tapping mode AFM that drives the oscillating probe tips in a sinusoidal manner to tap the sample surface periodically and record a series of force curves for each pixel point on the sample, where the maximum peak forces are maintained by tuning the vertical piezoelectric driver (z-piezo). The z-piezo movements of the probe are then be plotted as the sample 3D topography as a function of the *x*, *y* horizontal coordinates. The peak force tapping mode applies a fixed oscillation frequency that is far less than the probe cantilever resonant frequency and skillfully suppresses the long-range interaction forces, such as adhesive and electrostatic forces, leading to significant improvements in the image quality and fewer artificial errors derived from the complex tip-surface interactions and cantilever dynamics. All the features endow peak force tapping mode AFM with the unprecedented ability to precisely and simultaneously capture the height profiles and adhesive and stiffness maps of cell membrane pores, which is sufficient for the recognition of pore topography.

In the present study, we provide clear evidence that AFM, by virtue of its high resolution and noninvasive imaging capabilities, represents a useful tool to visualize pore formation in lymphocytes and tumor cells. The ability to visualize and understand pore formation is fundamental to improving our understanding of the basic processes of the immune clearance of pathogens and tumor cells, as well as pore formation-mediated cell death. Immune-related pore formation is mediated by perforin, the complement-induced MAC or gasdermins. Although the pores formed by different mediators exhibited different sizes and depths, AFM-mediated visualization actually reveals a dynamic pore formation process comprising pore fusion and repair. Overall, the present study revealed the crucial process of changes in the cell membrane structure that can lead to cell death.

## Materials and methods

### Cell lines

OVA-B16 (mouse melanoma), 293T (human embryonic kidney cell line), and MCF-7 (human breast cancer cell line) cells were purchased from the China Center for Type Culture Collection (Beijing, China) and cultured in RPMI 1640 medium (Thermo Scientific, Waltham, MA, USA) supplemented with 10% fetal bovine serum (FBS) (Gibco, Thermo Scientific, USA).

### Reagents and plasmids

SLO and PI were purchased from Sigma-Aldrich (St Louis, MO, USA). Pierce™ SATA (N-succinimidyl S-acetylthioacetate) and Pierce™ Hydroxylamine-HCl were obtained from Thermo Fisher Scientific. The anti-CD25 depleting antibody was purchased from Biolegend (SD, CA, USA). The anti-SLO antibody was obtained from Abcam (SD, CA, USA). The pCS2–3×flag-GSDME, Pgs2–3×flag-GSDMD and pLenti-NIrD-GSDMD-N(L192D)-eGFP plasmids were kindly provided by Dr. Feng Shao (National Institute of Biological Sciences).

### Sample preparation for AFM

Cells were seeded in 35 mm plastic plates with a glass insert. After treatment, cells were washed with PBS twice, fixed with 4% paraformaldehyde and imaged under the AFM.

### Atomic force microscopy

Force-distance curve-based AFM (FD-based AFM) was performed using a Dimension ICON AFM (Bruker, Santa Barbara, USA) set to PeakForce Tapping mode^32,33^. The AFM was equipped with a 90 μm piezoelectric scanner. The AFM cantilevers used in the present study (Bruker ScanAsyst-Air) had a nominal spring constant of 0.4 N m^−1^ and sharpened silicon tip with a nominal radius of 2 nm. The FD-based AFM topographs were recorded at room temperature, which ranged from 20–24 °C. The AFM was placed inside an acoustic isolation box and the maximum force applied to image the samples was 1 nN. The oscillation frequency and oscillation amplitude of the cantilever were set to 2 kHz and 50 nm, respectively. AFM images were analyzed and processed with Nanoscope Software (Bruker, Karlsruhe, Germany). We flattened each tapping mode image to measure the diameter and depth of every pore,. Pore diameters were measured from both the major axis and short axis around the pore. Pore depths were measured from the highest protruding rim relative to the lowest concave edge.

### Coupling the anti-SLO antibody to the AFM-tip

The AFM tips (NPG-10) were functionalized with the anti-SLO antibody according to the protocol published by Newton et al^34^., with some modifications. Briefly, NPG-10 tips were coated with the crosslinker NHS-PEG-maleimide for 1 h at room temperature and then rinsed three times. Simultaneously, the anti-SLO antibody was incubated with the SATA solution for 30 min at room temperature, followed by a 2 h incubation with a hydroxylamine solution at room temperature. Finally, the PEG-maleimide-coated NPG-10 tips were incubated with the thiol-functionalized antibody, leading to the binding of the anti-SLO antibody to the NPG-10 tips through the reaction of maleimide and the thiol groups.

### Scanning electron microscope (SEM)

OVA-B16 cells were cultured in a 24-well plate coated with silicon chips. After treatment, these cells were washed with PBS three times and fixed with 2.5% glutaraldehyde for 4 h. Then, cells were washed with 0.1 M phosphate buffer, further fixed with 1% osmium tetroxide (OsO_4_) for 1 h, and dehydrated by incubation with a graded series of ethanol solutions (50%, 60%, 80%, 90%, and 100%). After drying in critical point dryer with liquid CO_2_, cells were observed under Hitachi SU8010 SEM.

### PI staining

PI (100 μM) was added to the supernatant of cells for 30 s, and then these cells were collected, washed with PBS, and analyzed by flow cytometry.

### Plasmid transfection

For transient transfection, 293T cells were seeded in six-well plates. When the cells reached approximately 70% confluence, 293T cells were transiently transfected with Vec, pEGFP human-GSDMD-(1–275)N-terminal (2 μg/well) or pEGFP human-GSDME-(1–270)N-terminal (4 μg/well) for 18 h. Then, cells were imaged using AFM. A stable Tet-on pLenti-NIrD-GSDMD-N(L192D)-eGFP-expressing cell line was generated by lentiviral transduction and selection with blasticidin. Briefly, Pspa×2, pVSV-G and pLenti-NIrD-GSDMD-N(L192D)-eGFP or empty vector were transfected into HEK 293T cells using the FuGENE HD transfection reagent (Roche, Basel, Switzerland). Polybrene was mixed with the virus-containing supernatants to a final concentration of 8 μg/ml before addition to MCF-7 cells. MCF-7 cells expressing the Tet-on GSDMD-N(L192D)-eGFP plasmid were cultured with 5 μg/ml blasticidin and 10% FBS.

### Isolation of cytotoxic granules from CD8^+^ T cells

Cytotoxic granules containing PFR were isolated as previously described^35^. Briefly, blood was obtained from healthy donors, and CD8^+^ T cells were isolated by RosetteSep (Stem Cell Technologies, Vancouver, British Columbia, Canada), activated with anti-CD3/CD28 beads and maintained in RPMI 1640 medium supplemented with 10% FBS and 50 U of rIL-2. After one week of culture, cells were restimulated with another dose of anti-CD3/CD28 beads. After two additional weeks of incubation, the T cell blasts had reached a density of approximately 3 × 10^9^ total cells and were harvested. Then, T cells were homogenized and nonlysed cells, nuclei and other heavy cytoplasmic debris were removed. The final postnuclear supernatant was centrifuged at 15,000 × g for 10 min to pellet granules and heavy organelles. The pellet was then resuspended and carefully layered on top of a preformed continuous gradient of 40% adjusted Percoll and subjected to centrifugation at 48,000 × g for 10 min. The cytotoxic granules formed a visible refractile layer that was extracted and then subjected to an overnight centrifugation step at 64,000 × g to pellet and remove the remaining Percoll. Using PI staining, one unit of PFR was defined as causing 50% of OVA-B16 cells to be stained with PI after an incubation at 37 °C for 10 min.

### Immunofluorescence staining

SLO was conjugated with biotin using an EZ-link sulfo-NHS-LC biotinylation kit (ThermoScientific). OVA-B16 cells were cultured in 35 mm glass-bottom cell culture dishes for 24 h and treated with biotin-conjugated SLO (biotin-SLO). Then, cells were fixed with 4% paraformaldehyde, blocked with 5% BSA and incubated with Alexa Fluor 647-conjugated streptavidin (Invitrogen, Carlsbad, CA, USA) for 1 h at room temperature. An Olympus laser scanning confocal microscope (FVI000MPE) was used to image the cells.

### Complement depletion

CD4^+^CD25^+^ Treg cells were sorted from the spleens of C57BL/6 mice using FACS. Then, these Tregs cells were cultured in the presence of IL-2 (100 U/ml) for 4 h. Meanwhile, mouse plasma was isolated from the orbital of C57BL/6 mice and added to the culture medium of Treg cells. Afterwards, Treg cells were treated with an isotype control or anti-CD25-depleting antibody (PC-61, Biolegend, SD, CA, USA) for 5, 10, or 30 min. For some Treg cells, the anti-CD25 antibody was removed from the culture medium after 10 min.

### Statistical analysis

All experiments were performed at least three times. The results are presented as the means ± s.e.m and were analyzed using one-way ANOVA followed by Bonferroni’s test. A *p* value < 0.05 was considered statistically significant. All data met the assumptions of the tests. The analysis was conducted using GraphPad 6.0 software.

## Electronic supplementary material

supplemental information
