# Supplementary material for: Visualization of perforin/gasdermin/complement-formed pores in real cell membranes using atomic force microscopy
Source: Cell Mol Immunol. 2018 Oct 3;16(6):611–20. doi: 10.1038/s41423-018-0165-1 (PMC6804747; doi:10.1038/s41423-018-0165-1)
Supplement: Supplementary file 1 — supplemental information [file 41423_2018_165_MOESM1_ESM.docx]

**
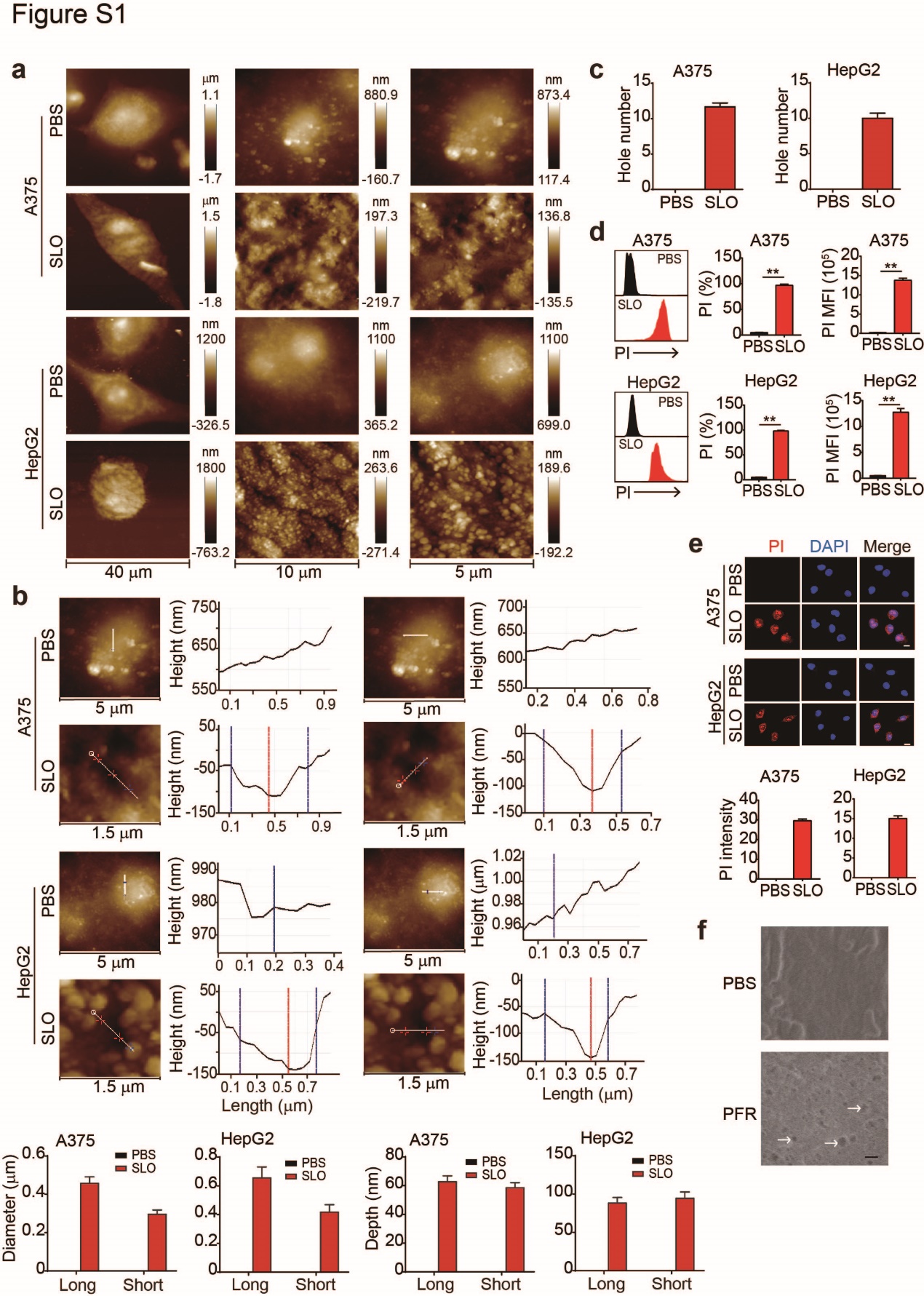
**

**Supplementary Figure 1 Pores formed by SLO are imaged by AFM and SEM. (a-c)** A375 or HepG2 cells were treated with SLO (50 U) for 15 min. The cells were fixed and imaged by AFM. (**a**) Representative AFM topographies. (**b, c**) The pore diameter and depth were measured (**b**). And the pore number was calculated by the section analysis on three areas of 5×5 μm^2^ from one cell (n=6) (**c**). (**d, e)** A375 or HepG2 cells were treated with 50 U SLO for 5 min. At the last 30 seconds of SLO treatment, PI (100 μM) was added into the culture medium and PI^+^ cells were analyzed by flow cytometry (**d**). Some cells were fixed and observed under confocal microscope (**e**). Bar, 20 μm. (**f**) OVA-B16 cells were treated with SLO (50 U) for 10 min, and then imaged by scanning electronic microscopy. White arrow indicates the pores. Bar, 100 nm. ** p<0.01, by student’s t-test (**d**). The data represent mean ± SEM of three independent experiments.

**
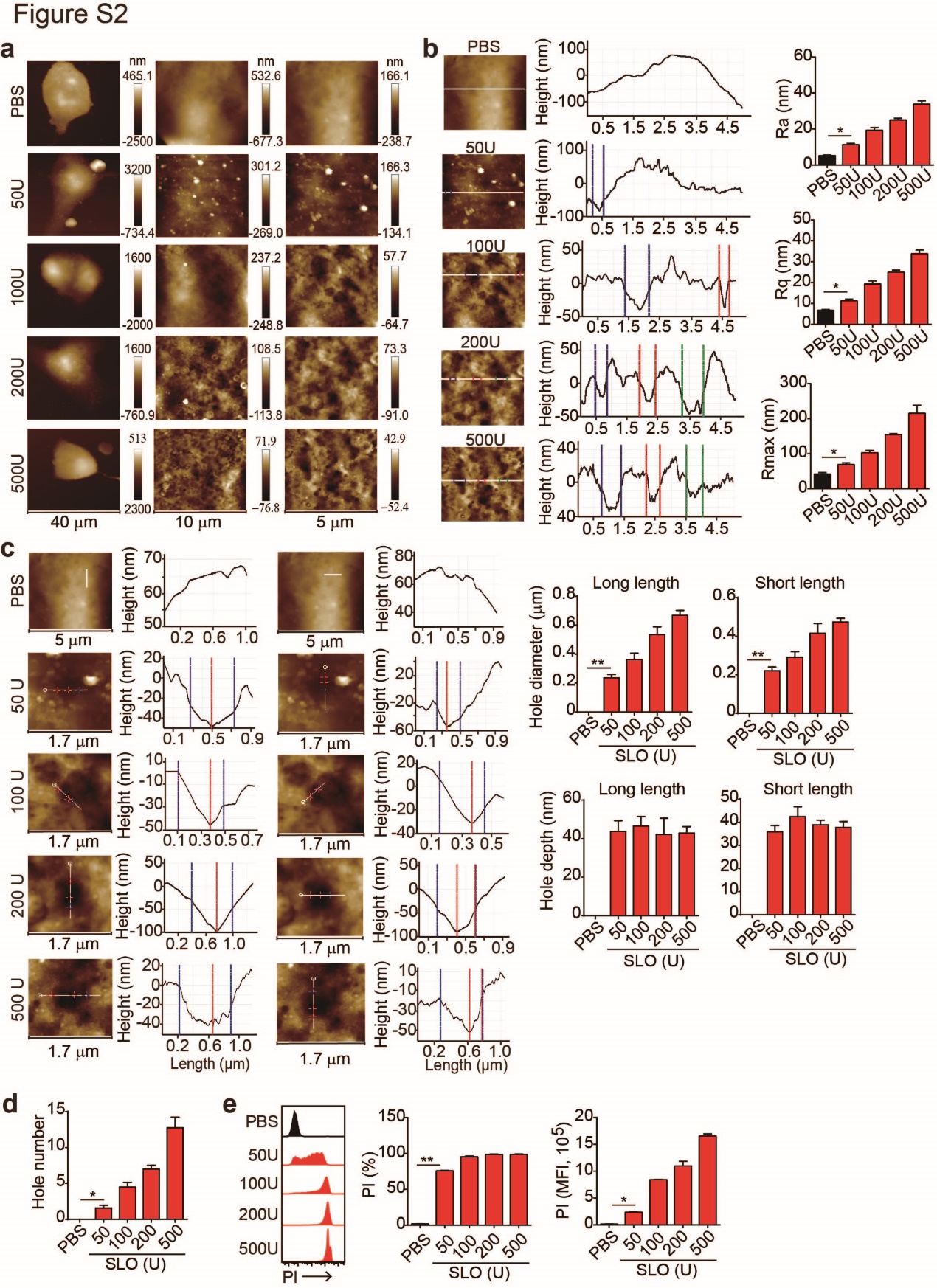
**

**Supplementary Figure 2** **SLO-mediated pore formation is dose dependent.** OVA-B16 cells (1×10^5^) were treated with different concentrations of SLO as indicated for 4 min. Then, the cells were fixed and scanned by AFM. (**a**) Representative OVA-B16 topographies under different amplification. (**b**) Surface roughness was analyzed, and the value of Ra, Rq and Rmax was calculated. (**c**) Section analysis of high-resolution AFM topographies. The pore diameter and depth were measured. (**d**) The number of pores was counted within 3 areas of three 5×5 μm^2^ from one cell (n=6). * p<0.05, ** p<0.01, by 1-way ANOVA (b-e). The data represent mean ± SEM of three independent experiments.


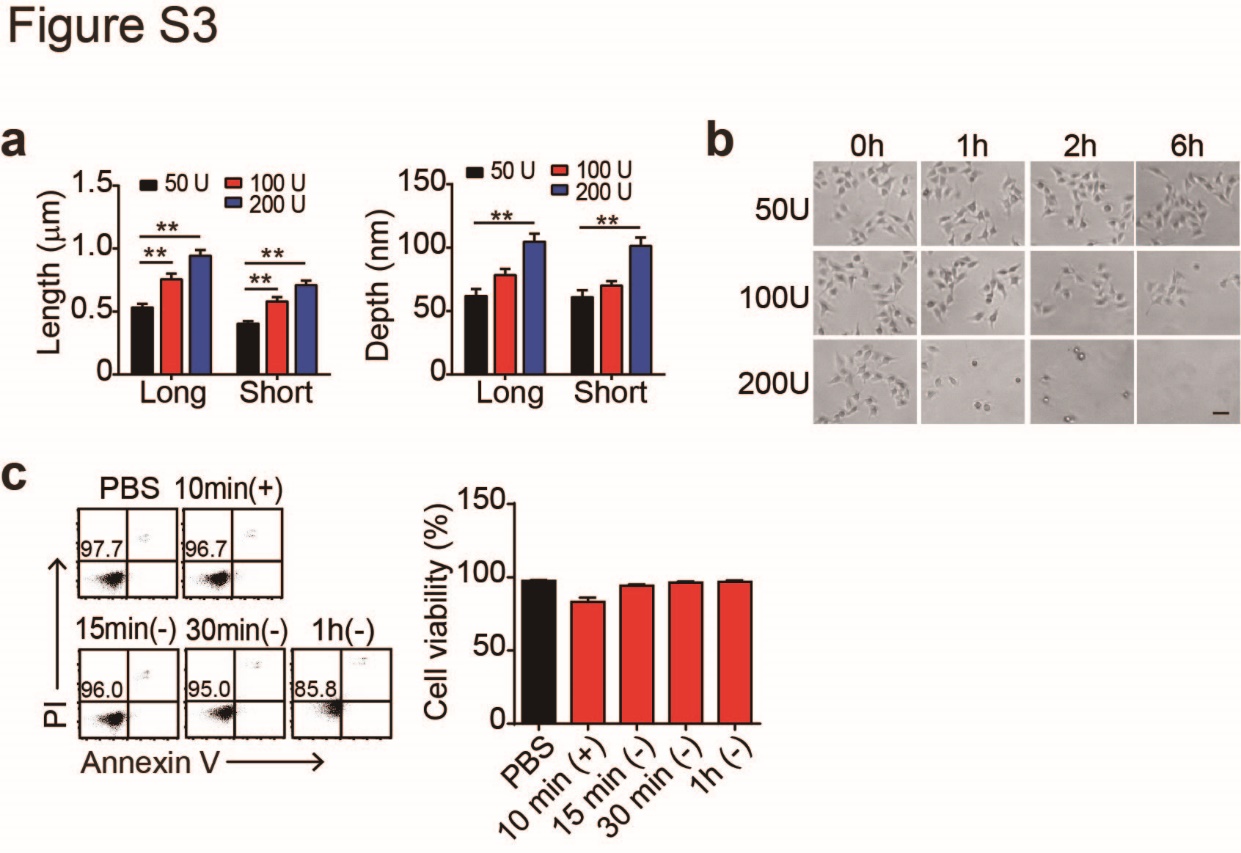


**Supplementary Figure 3 SLO-mediated pore formation is time dependent.** (**a**) OVA-B16 cells were treated with different doses of SLO for 10 min, and then imaged by AFM. The pore size was measured. (**b**) OVA-B16 cells were treated with SLO (50 U) for 10 min, and then cultured in fresh medium without SLO for another 1h or 2h. Then, these cells were imaged under light microscope. Bar, 50 μm. (**c**) OVA-B16 cells were treated with SLO (50 U) for 10 min, and then cultured in SLO-free medium for another 15 min, 30 min or 1h as indicated. Flow cytometry was used to determine the cell viability by immunostaining of Annexin V and PI. ** p<0.01, by 1-way ANOVA (a, c). The data represent mean ± SEM of three independent experiments. See also Figure 3.


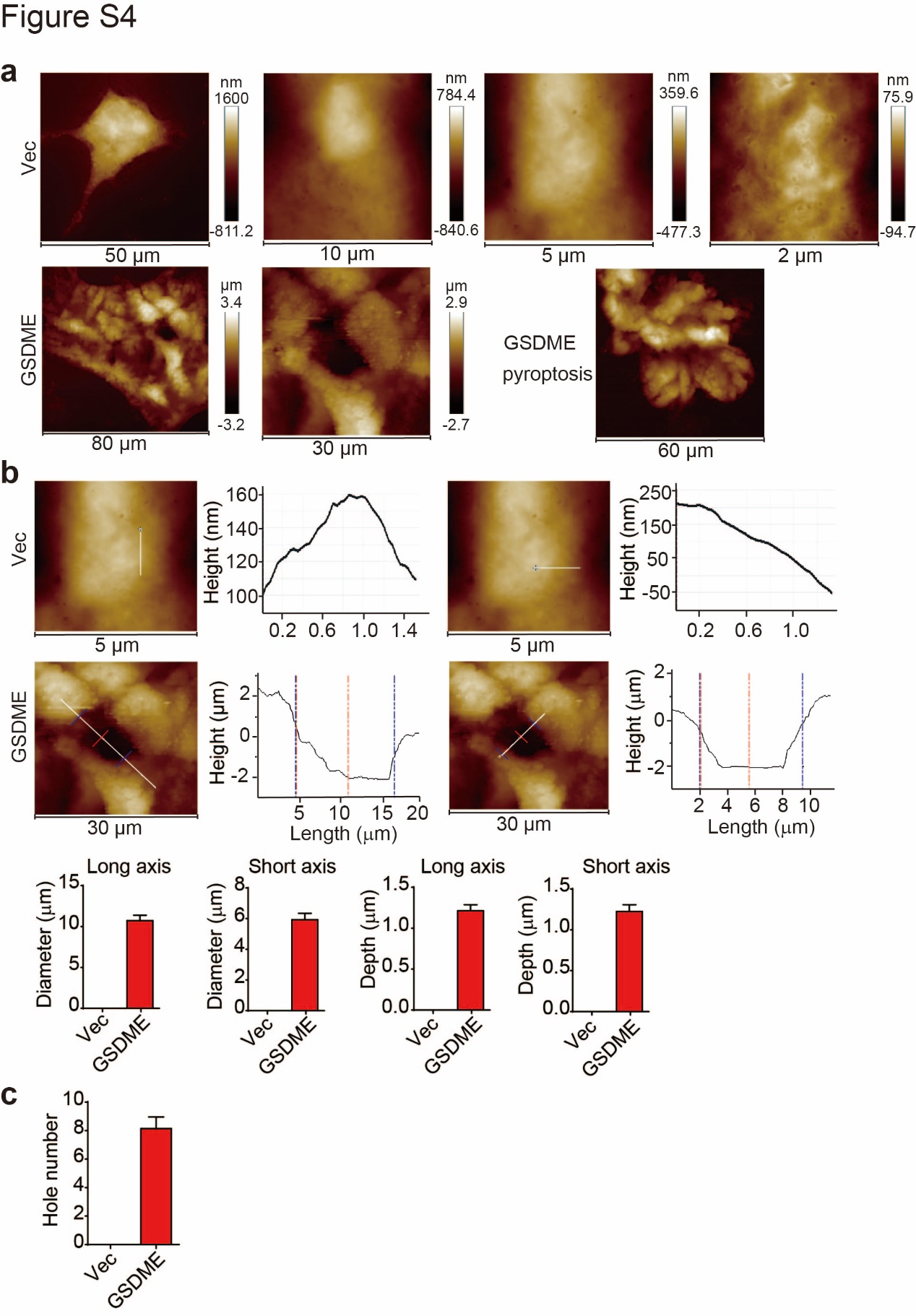


**Supplementary Figure 4** **Expression of the active form of GSDME leads to the pore formation in the membrane of 293T cells.** 293T cells were transiently transfected with Vec or GSDME^Nterm^ plasmid for 18h. The overexpressing GSDME cells were selected by fluorescence microscopy and further scanned by AFM. (**a**) Representative topographies of the outer plasma membrane in Vec or GSDME transfected cells were visualized by AFM. (**b**) Section analysis of the pore diameter and depth from high-resolution AFM topographies. (**c**) The number of pores was enumerated on 3 areas of 5×5 μm^2^ from one cell (n=6).
